# How does collegial support increase retention of registered nurses in homecare nursing agencies? a qualitative study

**DOI:** 10.1186/s12912-016-0157-3

**Published:** 2016-06-02

**Authors:** Maiko Noguchi-Watanabe, Noriko Yamamoto-Mitani, Yukari Takai

**Affiliations:** Gerontological Home Care and Long-term Care Nursing/Palliative Care Nursing, School of Health Sciences and Nursing, Faculty of Medicine, The University of Tokyo, Hongo 7-3-1, Bunkyo-ku, Tokyo, 113-0033 Japan; School of Nursing, Gunma Prefectural College of Health Sciences, 323-1 Kamioki-machi, Maebashi, Gunma 371-0052 Japan

**Keywords:** Collegial support, Homecare nursing agency, Qualitative research, Registered nurse, Retention, Turnover

## Abstract

**Background:**

Collegial workplace support has been linked to higher registered nurse (RN) retention in various clinical settings. In Japan, homecare agencies experience high RN turnover. The purpose of this study was to develop a conceptual framework to describe how perceived support from colleagues affects RNs’ decision to remain in an agency.

**Methods:**

A qualitative research method based on grounded theory was used. Participants were RNs with at least 4 years of experience at the same homecare agency. Participants were theoretically sampled via managers of 12 homecare nursing agencies. Semi-structured interviews and supplementary participant observations were conducted. Data were analyzed using a constant comparative technique, and the process of how workplace support affected participants’ decision to remain at their agency was clarified.

**Results:**

In total, 26 RNs were interviewed, 23 of whom were observed in their practice setting. Participants’ perception of support from colleagues was framed as being “*encouraged to grow in one’s own way*”, which comprised *practicing with protected autonomy* in an *insight-producing environment*. Participants reported that they were able to *practice with protected autonomy,* receiving 1) *mindful monitoring*, 2) *semi-independent responsibility*, 3) *help as needed*, and 4) *collegial empathy and validation*. RNs also felt supported in an *insight-producing environment* by 1) *enlightening dialogue*, 2) being *set for one’s next challenges,* and 3) being able to *grow at one’s own pace*. Reportedly, these were defining characteristics in their decision to continue working in their agencies.

**Conclusions:**

For RNs to willingly stay at a homecare nursing agency, it is essential that they are able to practice with protected autonomy in an insight-producing environment that encourages them to grow in their own way. Further research is needed to explore ways to create and enhance such environments to lower RN turnover.

## Background

In Japan, there has been a significant shift from hospital- to community-based healthcare systems, and more attention is being directed to homecare services. Homecare nursing plays a crucial role in caring for older adults in their own homes. However, homecare nursing agencies often experience serious registered nurse (RN) shortages. High RN turnover exacerbates these shortages, and it reduces an agency’s effectiveness and productivity [1] and the quality of care [2]. It has been reported that the average RN turnover rate for homecare agencies was 15.0 % per year [3], a rate higher than that of hospital nurses (11.0 %) [4]. Reducing this high turnover is therefore a pressing issue.

Most studies on nurse turnover have focused on hospital settings. The causal turnover model [5] is the most common theoretical model used to explain turnover. This model integrates 11 turnover-related factors including work environment and job satisfaction. However, studies have shown that this model accounted for only about 10 % of the variance in actual turnover [6, 7]. This suggests that an alternative model of turnover may better explain why nurses leave their workplace.

Little research is available on RN turnover in homecare settings [8]. Ellenbecker and colleagues [9] proposed a model of job retention for homecare nurses that examined 1-year retention, using factors such as intent to stay, job satisfaction, job tenure, and high wages. However, this model did not sufficiently explain actual turnover [10]. A qualitative study identified categories affecting turnover, such as work structure, staff relationships/communication, and work environment [11]. However, this study only listed those categories; it did not describe the relationship among them or how they contributed to RN retention. Greater understanding of how these categories contribute to RNs’ decision to remain in an agency is still needed.

Recently, literature has highlighted the importance of workplace support from colleagues (i.e., nurse managers and nurse coworkers) [12–14]. Research on magnet hospitals has emphasized the importance of the relationship among coworkers [15, 16]. Similar findings have also been recently reported in the homecare setting [11]. Exploring the types of support that are effective for RN retention may help developing the necessary policies and programs.

In Japan, homecare nursing agencies mainly employ RNs and some rehabilitation professionals [4, 17], with 4.7 RNs employed in an agency on average [4]. RNs working in homecare agencies generally have experience working as a RN in hospitals or health care facilities [18]. A wide variety of care, including infusion, palliative care, and family support is provided by homecare nurses [15]. Such agency characteristics should be considered when examining workplace support from colleagues in homecare nursing agencies.

This paper is part of a larger study that aimed to examine why RNs continue working at the same agency. The present focus is on describing RNs’ perceptions of how collegial workplace support affected their decision to remain with the agency. We used the term “colleague” to describe both nurse managers and RN coworkers.

## Methods

### Design

Grounded theory was used as it is suitable for developing theories and extracting categories to explain complex phenomena [19]. We used constant comparison of data, memo writing, and theoretical sampling, all of which are common to the multiple variants of grounded theory.

### Participants

Participants were staff RNs working in homecare nursing agencies, recruited from those with at least 4 years of experience in the same agency. Approximately 20 % of RNs quit their jobs in less than 4 years [3] and we intended to interview RNs with substantial work experience at one agency. The nurse managers of 12 homecare nursing agencies discussed this study with targeted staff RNs. If those RNs were interested in participating, the present researchers contacted them and explained the study in detail, including its voluntary nature. Written informed consent was obtained from all participants before their interviews. Verbal informed consent was obtained from all persons present in the agency when the researchers conducted observation in that agency.

Participants were recruited gradually as data analysis progressed over a 1-year period. The data collection strategy used theoretical sampling, and specific criteria were employed for agency recruitment: (1) recommended as providing high-quality care by one of four expert homecare nurses who had extensive (>10 years) experience working as a nurse manager at a homecare agency, and (2) a turnover rate less than 10 % in the past year. Each nurse manager recommended two RNs with different backgrounds to allow the researchers to determine if certain characteristics of communication belonged to the agency or the nurse. As the study progressed, we purposefully recruited participants who had past experience of quitting other agencies. This gave us a comparative view of the agency the nurse had left and his/her current workplace.

In total, 26 RNs were recruited from the 12 homecare agencies (Tables 1 and 2). Eleven homecare agencies mainly provided care for older adults, and one agency only provided care for children with disabilities. Despite these differences in client-base, there were no differences in participants’ demographic characteristics or interview and observation data.

**Table 1:** Participant characteristics (*N* = 26)

Characteristics		Mean ± SD N (%)
Age		42.2 ± 6.6
Sex	Female	25 (96)
Race	Asian	26 (100)
Employment status	Full-time	19 (73)
	Part-time	7 (27)
Job tenure at agency (years)		6.3 ± 3.6
Job experience in homecare nursing (years)		7.6 ± 4.1
Job experience (excluding homecare nurse agency) (years)		10.2 ± 5.9
Number of workplaces ever worked		4.0 ± 1.5
	Hospital	2.2 ± 1.2
	Homecare nurse agency	1.4 ± 0.8
	Other	3.8 ± 0.6
Partner		16 (62)
Children under 12 years old		6 (23)
Children		15 (58)

**Table 2:** Agency characteristics

		*N* = 26
Characteristics		Mean ± SD N (%)
Ownership	Profit	13 (50)
	Doctors organization	6 (23)
	Healthcare corporation	3 (12)
	Foundation	2 (8)
	Non-profit	2 (8)
Area	Urban	20 (67)
	Rural	6 (23)
Number of nurses	Total	10.7 ± 3.8
	Full-time	6.3 ± 2.5
	Part-time	4.3 ± 3.7
	Fulltime Equivalent	7.3 ± 2.4
Number of clients (per day)		145.8 ± 70.3

### Data collection

Data were collected through semi-structured interviews and participant observations between October 2012 and August 2013. In total, 26 semi-structured interviews were conducted; focusing on what encouraged participants to continue working at their agency. The interviews began with the question: *“Would you tell me how you started working at this agency?”* As the interview progressed, we asked *“In what kind of workplace would you want to continue working?”* This type of question allowed us to determine significant elements that RNs considered important for their continued work at an agency. Each participant was individually interviewed once by the first author, with interviews lasting 40–90 min. Interviews lasted 80–90 min, except for one interview that lasted 40 min owing to the participant’s time constraints. All interviews were audio-recorded. Field notes were taken after the interviews to record the researcher’s general impressions and immediate reflective thoughts.

Before the interviews, the first author, who had experience working as a homecare nurse, conducted participant observations. This was done to gain first-hand insight into the activities and work environments of each agency, and gather data on actual collegial support for participants. In the interviews, questions were asked about the observed support.

Field notes were taken concurrently with the observations. Of the 26 RNs interviewed, 23 consented to be observed in their practice setting. Three RNs did not consent to observation due to privacy reasons. In total, 24 h of observation was carried out.

### Data analysis

Constant comparative techniques were used in the data analysis [19]. All interview transcripts and field notes were typed out verbatim. Transcripts and field notes were read carefully and coded according to the meaning of the content. Multiple codes were sorted and grouped on the basis of multiple comparisons, and developed into categories [19]. Properties and dimensions of these categories were identified and used to deepen the understanding of categories and relationships among them. Over time, some categories were replaced by new categories that represented the data more accurately, and the relationships among categories were developed into a conceptual framework.

Throughout the data analysis, memos were made and diagrams were drawn to capture thoughts, insights, and ideas about the data and the process of analysis. Memos were written records of analyses and diagrams are visual devices that depict relationship among analytic concepts [19]. If a diagram did not seem to work, we returned to the data and searched for alternative representations that better fit the data.

Charmaz’s framework was used to ensure the quality (credibility, originality, resonance, and usefulness) of the grounded theory study [20]. Data triangulation (interview and observation) and self-reflective memos were used to ensure credibility and originality. Member checking, peer debriefing, and an external auditor were used to ensure the credibility, resonance, and usefulness of the present study. Member checking was conducted by asking participants to read the report and comment on whether they felt that the findings accurately reflected their experiences. The feedback suggested that the analyses suited their views. The first author made frequent memos concerning her reflections and feelings to minimize researcher bias, especially as she had experience of working as a homecare nurse. During the entire study there were continuous discussions among researchers with experience in grounded theory research and individuals with experience of working as RN.

## Results

The interviewed RNs’ perception of working at the homecare nursing agencies was framed as “*encouraged to grow in one’s own way*.” This meant that the RNs felt that they could practice with a certain amount of autonomy while being well-protected by their supervisor/seniors. This semi-autonomous practice enabled them to develop their style of providing care as well as prompting insights or discovery into the meaningfulness of their own care. The accumulated experience of discovery was often sufficiently exciting to motivate them to continue working at the same agency. Being “*Encouraged to grow in one’s own way”* comprised two categories: *practicing with protected autonomy* in an *insight-producing environment.*

### Practicing with protected autonomy

This category encapsulated the RNs’ feeling that their colleagues protected them from the risk of making mistakes and receiving excess imputation, while allowing them the maximum autonomy deemed appropriate for their current competence level:

*I want to work at an agency where the colleagues watch, accept, and allow me to do things in my own way—as long as what I do fits the manager’s standards, (the colleagues) would just watch me with gentle patience. Yet if I make a mistake in front of clients and their families, I know they (colleagues) would try to protect me and apologize with me. (ID 1)*

*Practicing with protected autonomy* included four components. RNs perceived that they were given: 1) *mindful monitoring* of their practice from their colleagues; 2) *semi-independent responsibility* for the client while being monitored; 3) *help as needed*; and 4) *empathy and validation* for their care. Each component is detailed below.

#### Mindful monitoring

In the interviews, RNs talked about how their colleagues would willingly hear details of their care, continuously assess their competence level, and follow up the outcomes of their performance. In the homecare setting, RNs usually visit a client’s home by themselves, and nobody knows what occurs in the client’s home other than the RN who actually visits that client. In the participating agencies, after returning from client visits, RNs talked in detail about what they did at a client’s home with their colleagues. By doing so they gained feedback from their colleagues and determined if their care was appropriate.

In the observations at the agencies, a commonly observed behavior by experienced RNs was active listening to the less experienced RNs. This allowed less experienced RNs to describe the clients and families they visited and what they did as RNs in detail. This talking–listening was observed in casual conversations among RNs and in formal meetings. Through these conversations, more experienced RNs constantly taught less experienced RNs about good and appropriate nursing care in home visits.

In situations where listening was not sufficient to allow experienced RNs to evaluate the care situation, they visited the client’s home along with the less experienced RN, often voluntarily. For example, a colleague watched how a RN new to homecare assessed severity of pain, jointly formed a plan with that RN, and comforted the client:

*(When) I talked about a client whose pain was constantly unbearable, (the manager) said “Well, I’ll go with you” and (the manager) came with me…(The manager) examined the client head to toe and also checked his living environment… (ID4)*

#### Semi-independent responsibility

When the experienced RNs considered a RN new to homecare could develop and implement a reasonable care plan, the client’s care was entrusted to that nurse. The care plan might not be exactly the same as a plan experienced RNs might develop, but it was allowed, provided it was within an acceptable range. The following example shows how a manager considered that judgment should be made by the staff RN once the client was entrusted to her:

*(The manager asked a nurse) “Do you think it’s possible to remove his (the client’s) mitten (to avoid facial and body injury?)” (The nurse answered) “It’s possible while I’m with him.” (Field note ID 23)*

RNs new to homecare were confident that their colleagues would point out anything inappropriate in their care plan. One RN reported:

*Well, I guess I can try out my own way as much as I want, because someone at the agency is watching me and will let me know if I do something wrong. So I don’t have to worry and I can do things the way I feel is right. (ID 7)*

If a care plan was not satisfactory, experienced colleagues would offer suggestions. However, the decision about whether to follow a suggestion was left up to the RN.

#### Help as needed

Even when RNs do their best, some difficult situations may arise, such as clients/families complaining or refusing nursing services. When this occurred, the interviewed RNs were confident that their colleagues would not criticize them, trusting that the RNs had done their best for the clients/families. The RNs also knew that if necessary, their colleagues would fully support them and provide assistance. Therefore, RNs felt secure in extending the best possible care to clients/families.

For example, a participant talked about an instance where she attempted food intake for a client at risk of aspiration. The client and family insisted on eating even though there was a risk of aspiration, but the RN needed to have confidence in her manager’s support, so she could allow the client/family to eat:

*I knew that the manager would support me and stand for me if something (an adverse event such as aspiration) would happen. That was why I could provide my best care for the client (assisting the client with food intake) without feeling insecure…Fortunately, this client had no trouble. (ID 11)*

Even if the RNs had not personally experienced this type of situation, they saw and heard what happened to other RNs in that agency. They developed a sense of trust that their colleagues would support them, as they accumulated such experiences:

*I absolutely trust the manager that if something happens (she would help me). If I make a mistake, I am sure the manager would apologize with me. I am very confident that the manager would handle the situation with me, definitely. (ID1)*

The RNs felt that their colleagues would support them in front of staff from other agencies when necessary. Where RNs work with a variety of other professionals in the community, some conflict may occur and RNs may receive criticism from others. On such occasions, the participating RNs knew their colleagues would support them and help them feel that they were not alone:

*When someone was critical of me at a service meeting, my manager said “I’m sure she (the interviewed nurse) didn’t make a mistake. I trust her. But if she did make a mistake, I am responsible for that.” It was truly impressive and I thought “Wow! (My manager really protects me!)” (ID3)*

#### Collegial empathy and validation

The interviewed RNs recognized that their colleagues helped them to express their feelings. As RNs, they might experience feelings of hurt from a client’s words or attitudes; on such occasions, they felt relieved by their colleagues’ actively listening to and sympathizing with them:

*Sharing feelings, worrying together, and crying together—and then, I don’t feel I have to carry the burden alone (ID16).*

In the interviews, many RNs verbalized anxiety about their everyday practice, because in homecare nursing, unlike hospital nursing, they are by themselves in making decisions and providing nursing care. They felt reassured about their practice when their colleagues listened to what they did for a client and validated it. RNs new to homecare are sometimes not confident about how their care contributes to their client’s life. This is because unlike hospital nursing, the client’s status does not necessarily improve and things may not change. The experienced RNs offered reinforcement that the RN’ care maintained the client at home in a stable condition, and that it is the power of home nursing care:

*What is the effect of my nursing care? Sometimes, I worry—but my colleagues told me that what I provided was the best care for the client at that time. (ID15)*

In practicing with protected autonomy, the interviewed RNs felt secure about their practice and continued working at that agency. In addition to *practicing with protected autonomy*, RNs also perceived a second category of support: being able to practice in an *insight-producing environment*.

### Insight-producing environment

The participating RNs indicated that they were given many subtle cues by their colleagues leading to new insights, discovery, and growth as a nurse. This workplace environment was named an *insight-producing environment*. An insight-producing environment was created for staff RNs by 1) being involved in *enlightening dialogue,* 2) being *set for one’s next challenges*, and 3) being allowed to *grow at one’s own pace*.

#### Enlightening dialogue

The interviewed RNs reported that they had ample opportunity for dialogue with agency colleagues in both formal meetings and informal chats. Asking questions and talking freely with colleagues induced new awareness and perspectives that the RN had not previously realized. For example, when a RN returned from a visit in late afternoon, dialogue occurred naturally:

*A nurse came back from a home visit. Other nurses came back from home visits soon after. One nurse asked “How was today’s home visit?” Three nurses gathered naturally and shared their experiences. (Field note)*

Dialogue and questioning occurred frequently in all of the observed agencies. In these dialogues, RNs heard about new perspectives, reconsidered their care plans, or recognized a previously overlooked client’s response:

*(A nurse talked about a client with incontinence) “I cannot control leakage of urine with an incontinence pad…” Then another nurse said “Oh, why not use two incontinence pads? Two pads are OK for him (the client).” The nurse said “I didn’t think about it. I’m going to try.” (Field note)*

Overhearing other RNs’ conversations were reported to be as informative as direct discussion, especially if multiple RNs were in charge of the same client and more than two RNs had visited the client’s home together. RNs could recognize a client’s need and make changes after hearing other RNs’ conversations:

*I had never noticed or thought about this point until I heard other nurses talk about this client with the manager. (ID23)*

#### Set for one’s next challenges

Despite busy schedules, the interviewed RNs reported that they were occasionally given opportunities for new challenges, such as taking a training course or undertaking some research project. For example, a RN with less experience in discharge support was offered the chance to take charge of a discharge assistance case as an opportunity to grow. Similarly, the interviewed RNs reported that their managers sometimes intentionally changed the clients they had main care of, with the purpose of having these RNs gain diversity in their experiences:

*Most clients of mine now need rehabilitation or have pressure ulcers—I have to take care of them, although I’m not too good at rehabilitation or pressure ulcer care now—I guess my manager has assigned me these clients on purpose. (ID 7)*

RNs reported that they often were assigned to different clients, and this usually happened when they were feeling less satisfied with their job. This may have been the senior RNs’ consideration to give diversity to their experience. A senior RN who was in charge of client assignment said:

*I try to add changes to their home visits. Diversity in everyday practice should be provided for everyone—and for me. (ID 13)*

#### Grow at one’s own pace

After receiving the seeds of a challenge, the RNs were given time to decide whether they would take that challenge. For example, when a RN was asked whether he/she would serve as a mentor for a newcomer, that RN was given enough time to decide:

*In the meeting, a nurse said “I’d like to serve as a mentor for a newcomer; the manager asked me about it 2 weeks ago.” (Field note)*

The three above components created an *insight-producing environment*; this environment provided support for RNs to continue working at their agency.

## Discussion

This study focused on staff RNs’ perceptions of collegial support and how it affected their retention.

Participating RNs identified support that allowed them to practice with protected autonomy. A previous study with nurse managers found that skillful managers supported RNs to gain confidence and overcome feelings of uncertainty, and provided a bridge between a RN new to homecare and senior RNs so they could talk about their work experiences [21]. Some of our interview findings are similar to the support methods discussed by managers in that study; interviewed staff RNs recognized that experienced RNs monitored their competency, provided help when necessary, and validated their practice. Accumulating such positive experiences fostered feelings of security that resulted in a staff RN continuing to work at the agency.

It was also suggested that RNs were willing to accept a certain level of responsibility for care and this would promote staff RN retention, providing that their competence level was monitored carefully. Previous studies found that a high degree of autonomy [22] and discretion [23] were factors associated with retention of hospital staff RNs. However, evidence on the importance of autonomy in the homecare setting is lacking. Autonomy was not included in the theoretical model of homecare nurse turnover [24]. The importance of staff RN autonomy may have been missed because most studies on homecare nurse turnover have focused on managers’ perspectives; there may be a disparity between a manager’s perception of factors important for RN retention and the perceptions of RNs who have left an agency [25]. Appropriate discretion may provide new insights for RNs, and may make their work more meaningful and interesting to them, which in turn may reduce turnover.

Our study emphasized the importance of support from coworkers as well as from managers. Staff RNs’ new insights were often generated from informal dialogue among coworkers. This is consistent with findings from studies in the business sector that support from coworkers, not managers, significantly influenced competency [26]. Some previous studies reported that coworkers’ support of practice and active listening to concerns were important for homecare nurses [27, 28]. As many studies on staff turnover have explored the perspectives of managers, the importance of open dialogue among coworkers might have been overlooked. This finding suggests that to promote staff RN retention, nurse managers should facilitate communication/dialogue among coworkers, for example, by setting regular meetings or gatherings.

Another important finding was that colleagues helped staff RNs to develop their own insight through promoting self-determination. Knowles reported that adult learners need to be involved in the planning and evaluation of their own education, so that they themselves understand how their learning program was developed and how they are to learn [29]. In our study, interviewed RNs perceived that their decisions regarding client care and their practice goals were respected by their colleagues, and this perception motivated them to continue working at that agency. This suggests that colleagues’ respect for a RN’s decisions helps them to remain in an agency.

### Limitations

A major limitation of our study was that we did not compare the interviews with RNs who stayed the study agencies and those who did not stay. This was because the study purpose was to explore how perceived collegial support affected the retention of homecare nurses. However, our purposeful recruitment of participants who had experience of quitting other agencies would have allowed us to compare differences between the agency they left and their current workplace to some extent. Further research is needed to explore RNs’ reasons for leaving an agency. Another possible limitation is that most participants were female. However, this limitation might not bias the findings as 94.4 % of Japan’s RNs are female [4].

We hope that our study makes a significant contribution in helping managers and senior RNs to promote retention of homecare nurses. In addition to providing quality work environments in terms of salary and the number of on-calls, collegial support may have significant implications for RN turnover [14]. Existing management textbooks describe the issue of staff management using abstract terms such as “establishing ideal working conditions.” [30]. The concrete and specific support methods described in our study may assist managers to work better with their staff RNs.

## Conclusion

This study found that support to “*grow in one’s own way”* from nurse colleagues promoted RN retention in homecare nursing agencies. One component of “*growing in one’s own way”* was *practicing with protected autonomy*, which includes *mindful monitoring*, *semi-independent responsibility*, *help as needed*, and *collegial empathy and validation* of their practice. Another component was an *insight-producing environment*, which included *enlightening dialogue*, being *set for one’s next challenges*, and being allowed to *grow at one’s own pace*. Further research is needed to explore how to increase the number of agencies that provide these types of collegial support.

## Abbreviations

RN, Registered nurse.
